# Leucine-Rich Repeat in Polycystin-1 Suppresses Cystogenesis in a Zebrafish (*Danio rerio*) Model of Autosomal-Dominant Polycystic Kidney Disease

**DOI:** 10.3390/ijms25052886

**Published:** 2024-03-01

**Authors:** Biswajit Padhy, Mohammad Amir, Jian Xie, Chou-Long Huang

**Affiliations:** Department of Internal Medicine, Division of Nephrology, Carver College of Medicine, University of Iowa, Iowa City, IA 52242, USAjian-xie@uiowa.edu (J.X.)

**Keywords:** polycystin-1 protein, polycystic kidney disease, leucine-rich repeats, laminin, zebrafish

## Abstract

Mutations of *PKD1* coding for polycystin-1 (PC1) account for most cases of autosomal-dominant polycystic kidney disease (ADPKD). The extracellular region of PC1 contains many evolutionarily conserved domains for ligand interactions. Among these are the leucine-rich repeats (LRRs) in the far N-terminus of PC1. Using zebrafish (*Danio rerio*) as an in vivo model system, we explored the role of LRRs in the function of PC1. Zebrafish expresses two human *PKD1* paralogs, *pkd1a* and *pkd1b*. Knockdown of both genes in zebrafish by morpholino antisense oligonucleotides produced phenotypes of dorsal-axis curvature and pronephric cyst formation. We found that overexpression of LRRs suppressed both phenotypes in *pkd1*-morphant zebrafish. Purified recombinant LRR domain inhibited proliferation of HEK cells in culture and interacted with the heterotrimeric basement membrane protein laminin-511 (α5β1γ1) in vitro. Mutations of amino acid residues in LRRs structurally predicted to bind laminin-511 disrupted LRR–laminin interaction in vitro and neutralized the ability of LRRs to inhibit cell proliferation and cystogenesis. Our data support the hypothesis that the extracellular region of PC1 plays a role in modulating PC1 interaction with the extracellular matrix and contributes to cystogenesis of PC1 deficiency.

## 1. Introduction

Autosomal-dominant polycystic kidney disease (ADPKD) is an inherited monogenic disorder diagnosed by renal cysts typically found in adults between the ages of 30 and 50. ADPKD is an important cause of end-stage renal disease (ESRD), and apart from kidney cysts, it may lead to extrarenal complications like development of cysts in the liver and pancreas, intracranial aneurysms, diverticulosis, and cardiovascular abnormalities [1]. The majority of ADPKD cases are due to mutations in the genes polycystic kidney disease 1 (*PKD1*) and polycystic kidney disease 2 (*PKD2*), which encode for polycystin-1 (PC1) and polycystin-2 (PC2) protein, respectively. PC1, mutations of which causing ~80% of ADPKD cases, is an integral membrane-bound protein localized to cilia, plasma membrane, desmosomal junctions, and endoplasmic reticulum (ER) [2,3,4]. It consists of 4303 amino acids containing a long extracellular N-terminus, 11-transmembrane domains, and a relatively shorter cytoplasmic C-terminus. PC2 is a Ca^2+^-permeable non-selective cation channel.

The last six transmembrane (TM) segments of PC1 interact with corresponding transmembrane segments of PC2 in a 1:3 stoichiometric ratio to form a PC1/PC2 heterotetramer polycystin channel complex [5]. The 226-amino-acid-long C-terminal tail of PC1 is shown to interact with multiple signaling proteins such as STAT6, P100 [6], heterotrimeric G-proteins [7], and AP-1 activation [8]. In addition, the cytoplasmic C-terminal tail of PC1 is found to interact with the coiled-coil domain of PC2, which may regulate PC2 channel activity [9,10].

The extracellular N-terminal region of PC1 contains more than two-thirds of the PC1 protein (>3000 amino acids). There are seven distinct, evolutionarily conserved structural domains in the extracellular region of PC1, most of which are reported to be involved in cell–cell or cell–matrix interactions [11]. Association of PC1 and PC2 may allow PC2 to respond to external stimuli through the extracellular N-terminal region of PC1. Disturbances of these interactions may lead to cystogenesis. However, the developmental and subcellular expression of PC1 and PC2 are not congruent [2,12,13,14]. Thus, PC1 may work independently of PC2. To elucidate the function of the extracellular region of PC1 in the progression of renal cystogenesis, we employed zebrafish (ZF) (*Danio rerio*) as an in vivo model system for ADPKD. We show that cystic phenotypes in PC1-knocked-down ZF embryos can be suppressed by transgenic overexpression of N-terminus leucine-rich repeats (LRRs). We provide evidence that this is likely though LRR binding to laminin to modulate laminin–integrin interaction. The results support the notion that the extracellular region of PC1 plays an important role in anti-cystogenesis.

## 2. Results

### 2.1. Zebrafish Human PKD1 Paralogs, pkd1a and pkd1b, Have Redundant Functions

**Figure 1:**
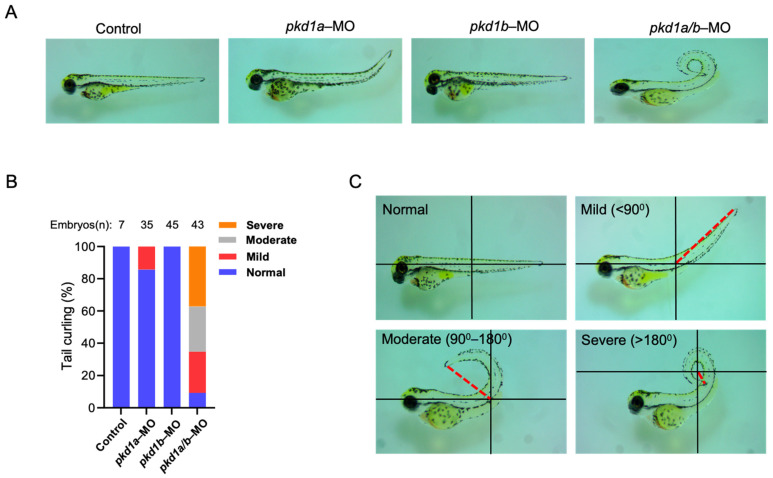
Knockdown of both *pkd1a* and *pkd1b* genes results in dorsal-axis curvature in zebrafish embryos. (**A**) shows uninjected control ZF embryos and embryos injected with either *pkd1a*, *pkd1b,* or both morpholinos at ~3 days post-fertilization (dpf). *Pkd1a* and *pkd1b* genes are shown to be redundant with no drastic effects when injected alone, while combined knockdown results in embryos with varying degree of dorsal-axis curvatures. Images are taken on a dissecting microscope at 4× magnification. (**B**) shows percentage of pkd1a/b-morphant ZF embryos segregated based on tail curvatures in groups injected with vehicle or morpholinos for *pkd1a*, *pkd1b*, or both. The total number (n) of embryos from combined multiple experiments is shown on top. (**C**) Images of ZF morphants deficient in both *pkd1a* and *pkd1b* based on the degree of dorsal body axis curvature at ~3 dpf. Dorsal body axis is represented by red dashed line in relation to the body axis. Embryos were segregated as normal (no curvature), mild (<90°), moderate (90–180°), and severe (>180°) based on the tail curvature.

To examine the role of PC1 in cystogenesis, we employed zebrafish as a model because of their ease of genetic manipulation and short life cycle. Zebrafish have two paralog genes to the human *PKD1*, *pkd1a* and *pkd1b*. Zebrafish with both genes knocked down exhibit phenotypes of dorsal-axis curvature and cysts in pronephric ducts [15]. Fertilized zebrafish embryos at 1 to 4 cell stage were injected with previously validated anti-sense morpholinos targeting either *pkd1a* or *pkd1b* [15]. As reported, *pkd1a* and *pkd1b* genes have overlapping and redundant functions, and knockdown of both was required for full exhibition of dorsal-axis curvature (Figure 1A–C). Around 14% of *pkd1a*-morpholino-injected embryos showed only mild dorsal-tail curvature (<90° relative to body axis) at ~3 days post-fertilization (dpf), while there were no detectable phenotypes in morphants injected with *pkd1b* morpholino (Figure 1A,B). Co-injection of both *pkd1a* and *pkd1b* morpholinos resulted in *pkd1a/b* morphants with a more severe dorsally curved body axis (Figure 1A–C). Due to dilution and uneven distribution of oligonucleotides among rapidly dividing cells, the severity of phenotypes varies among individual embryos. For phenotypic analysis, they were segregated into severe (>180°), moderate (90–180°), mild (<90°), and normal categories (no curvature) based on the degree of curvature (Figure 1C).

### 2.2. Overexpression of N-Terminal Leucine-Rich Repeats Suppresses Dorsal-Axis Curvature and Pronephric Cystic Phenotypes in pkd1a/b Morphants

**Figure 2:**
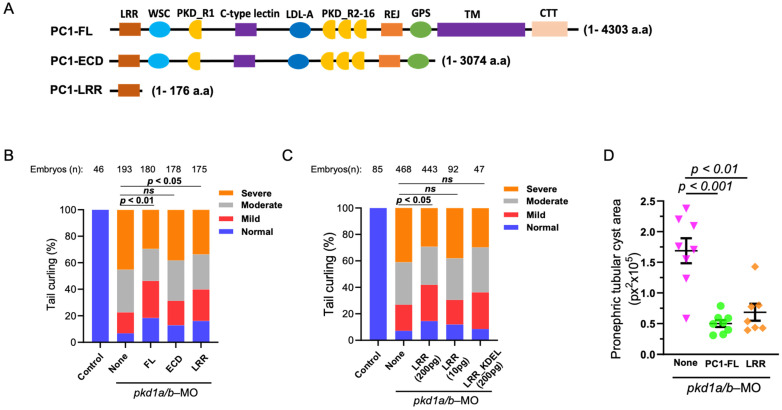
LRR suppresses cystic phenotypes in PC1-morphant zebrafish embryos. (**A**) Diagrammatic presentation of PC1 full-length (PC1-FL), truncated extracellular domain (PC1-ECD), and LRR (PC1-LRR) domain alone. (**B**) shows the percentage of pkd1a/b-morphant ZF embryos segregated based on severity of tail curling in groups injected with pkd1a/b morpholino along with vehicle (None) or mRNA expressing PC1-FL, ECD, or LRRs alone. Unlike ECD, co-injection of LRRs significantly rescued tail curvature like that of FL. Statistical significance was calculated by comparing the relative ratio of each category between groups by χ^2^ test. (**C**) Transgenic expression of the LRR domain rescued the tail curvature phenotype in pkd1a/b-morphant ZF embryos in a dose-dependent manner, while expression of ER-targeted LRR_KDEL mutant did not rescue the tail curvature phenotype. (**D**) Pronephric tubular cyst areas are quantitated in pkd1a/b-MO ZF embryos injected with pkd1a/b morpholino along with vehicle (None, n = 8) or mRNA expressing FL (*n* = 8) or LRR domain (n = 7). Cystic area is presented as pixels squared (px^2^). Data are presented as mean ± SEM. Data presented are from at least 3 independent experiments. The total number (*n*) of embryos from combined multiple experiments is shown on top. Statistical analysis was performed by χ^2^ test or *t*-test. ns = not significant.

We examined the role of whole extracellular N-terminus of PC1 and LRR in reversing PC1-deficient phenotypes (Figure 2A). mRNA coding for full-length PC1 (“PC1-FL”), the entire extracellular domain (“PC1-ECD”), or LRRs (“PC1-LRR”) was co-injected into zebrafish embryos along with *pkd1a* and *-b* morpholinos. Supporting that dorsal-axis curvature in pkd1a/b-morphant embryos is due to loss of PC1 function, re-expressing full-length human PC1 significantly suppresses dorsal-tail curvature (Figure 2B). However, tail curling in pkd1a/b morphants is not reversed by injecting an equal amount (200 pg) of mRNA expressing the ECD of PC1. Interestingly, co-injecting the same amount of mRNA expressing LRRs significantly suppressed dorsal-tail curvature in the morphant embryos. The amino acid sequence of FL and ECD (4303 and 3074 amino acids) is much longer than LRRs (176 amino acids). Since the same amount (200 pg) of mRNAs was injected for FL, ECD, and LRRs, the number of mRNAs and corresponding expressed protein molecules for LRRs may be as high as 25 times more than FL-PC1. We performed dose-dependent studies on the effect of LRRs on reversing PC1 deficiency. Injecting 1/20th of the amount of LRRs (10 pg) did not reverse dorsal curvature in pkd1a/b morphant as 200 pg of LRRs (Figure 2C). Expressing ER-targeted LRRs (“LRR-KDEL”) even at 200 pg had no effect on the suppression of tail curling in pkd1a/b morphants, indicating that extracellular secretion of LRRs is necessary for rescuing of tail curling in PC1-deficient zebrafish embryos.

In addition to dorsal-axis curvature, the severity of pronephric tubular cysts (Supplementary Figure S1) in pkd1a/b morphants was analyzed, which was previously shown to correlate with degree of curling (Figure 2D) [16]. Embryos injected with pkd1a/b morpholino alone (None) exhibited enlarged pronephric tubular cysts. In comparison, pkd1a/b morphants co-injected with mRNA for FL-PC1 or LRRs significantly suppressed pronephric tubular cyst area in agreement with rescue of tail curling.

### 2.3. LRRs Play an Anti-Proliferative Role in Cell Proliferation

**Figure 3:**
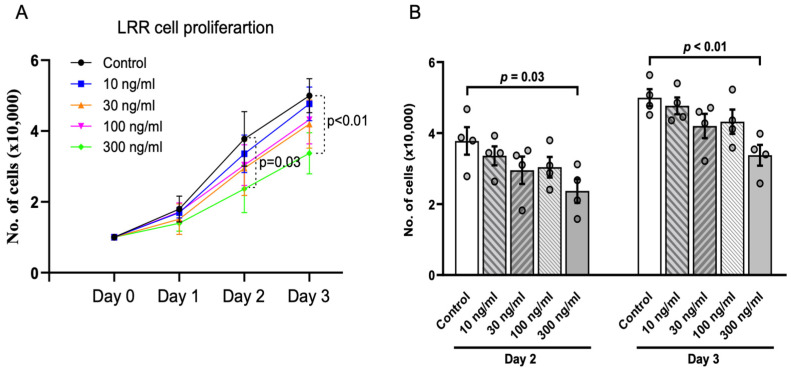
Anti-proliferative effect of LRR domain in vitro. (**A**) presents cell proliferation assays in HEK293 cells. Addition of purified LRR domain alone at 300 ng/mL significantly inhibited cell proliferation compared to control cells on Day 2 and 3. (**B**) Bar graphs showing inhibition of cell proliferation by LRRs is dose-dependent. Data shown are from at least 3 independent experiments. Data are presented as mean ± SEM.

So far, transgenic expression of the LRR domain in ZF embryos supports its anti-cystogenic effect by suppressing dorsal-tail curvature and pronephric cyst area. A previous study reported that LRRs play a suppressive role in cell proliferation [17]. We used HEK293 cells as an in vitro model to validate the effect of LRRs. Purified LRR domain was added to the culture media of proliferating HEK293 cells at different concentrations ranging from 10 ng/mL to 300 ng/mL (Figure 3A,B). At low dosage (10, 30, and 100 ng/mL), LRRs tended to decrease the cell proliferation rate compared to no LRRs, but the effects were not statistically significant. However, cell proliferation was significantly inhibited at 300 ng/mL LRR on Day 2 and 3. Thus, LRRs cause a dose-dependent inhibition of cell proliferation of HEK cells in culture.

### 2.4. LRR Binds to Extracellular Matrix Protein, Laminin-511

**Figure 4:**
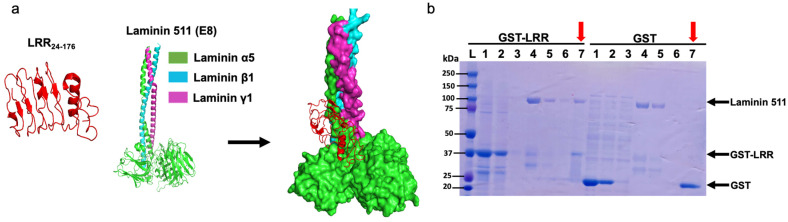
An LRR binds to an extracellular matrix protein laminin-511. (**a**) In silico analysis suggests a molecular interaction between the LRR domain and E8 fragment of heterotrimeric laminin-511 composed of laminin α5, laminin β1, and laminin γ1 chains. (**b**) Co-purification of laminin-511 along with GST-LRR suggests a physical interaction. GST-tag alone was used as negative control. M = protein marker, 1 = loading sample, 2 = flow through, 3 = wash, 4 = Lamin511 loading sample, 5 = flow through, 6 = washing of laminin after 1 h incubation, 7 (red arrows) = co-elution of GST-LRR or GST (or GST-LRR mutant E107A or W139C in Figure 5B) with laminin. Representative data shown are from 3 independent experiments.

Previous studies have shown that ECD of PC1 binds various extracellular matrix (ECM) proteins like collagen, fibronectin, and laminin [17,18]. Binding to these ECM proteins may modulate cell–cell and cell–matrix contacts, which can affect cell proliferation. We hypothesized that LRRs may interact with basement protein components, thereby regulating cell proliferation. Through in silico analysis, we found a molecular interaction between LRRs and E8 fragment of laminin-511, a heterotrimeric ECM protein that promotes cell proliferation (Figure 4a) [19]. E8 fragment of laminin-511 is composed of truncated C-terminal regions of α5, β1, and γ1 chains and is shown to be indispensable for integrin binding [20,21]. To validate the molecular interaction in vitro, we performed co-purification assays between GST-tagged LRR and commercially available laminin-511. As shown in Figure 4b, laminin-511 copurified with GST-tagged LRR domain (left panel, lane 7 indicated by red arrow) but not with the control GST-tag alone (right panel).

**Figure 5:**
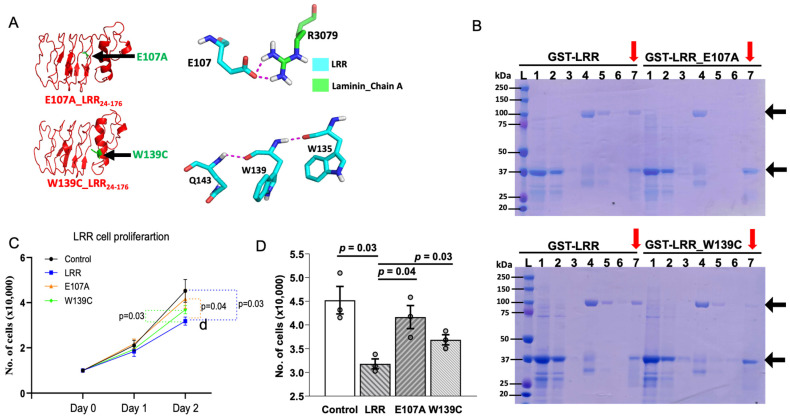
LRRs mediate the anti-proliferative effect by regulating PC1–laminin interaction. (**A**) (Upper panel) Structural analysis predicts that glutamate residue at E107 of LRR domain interacts with arginine residue at 3079 of α5 chain in laminin-511. Similarly, LRR residue tryptophan at 139 (lower panel), an ADPKD pathogenic variant, was found to be in a crucial α-helix conformation with Q143 and W135, alteration of which may hinder LRR–laminin interaction. (**B**) Unlike LRRs, laminin-511 was not copurified along with E107A and W139C mutants. M = protein marker, 1 = loading sample, 2 = flow through, 3 = wash, 4 = loading of Lamin511_Trimer, 5 = flow through, 6 = washing of laminin after 1 h incubation, 7 (red arrows) = co-elution of GST-LRR/GST/E107A/W139C and laminin. Representative data shown are from 3 independent experiments. (**C**,**D**) show cell proliferation is significantly inhibited by addition of LRRs (300 ng/mL) on Day 2 but not by E107A and W139C mutants. Data shown are from at least 3 independent experiments. Data are presented as mean ± SEM.

Molecular docking identifies that amino acid glutamate-107 (E107) and tryptophan-139 (W139) of LRRs may be important for interacting with laminin-511. E107 in LRRs interacts with arginine-3079 (R3079) of α5-chain in laminin-511 through hydrogen bonding (Figure 5A). W139 forms hydrogen bonds with glutamine-143 (Q143) and tryptophan-135 (W135) within LRRs, which is important for tertiary structure for interaction with laminin-511 (Figure 5A). To substantiate the findings of molecular docking, we examined LRR–laminin-511 interaction by mutating these amino acid residues in the LRR domain. In contrast to WT LRR, laminin-511 did not copurify with GST-tagged LRRs carrying the E107A mutation (Figure 5B, upper panel). Similarly, the LRR domain with the W139C mutation showed reduced interaction with laminin-511 (Figure 5B, lower panel). Moreover, application of LRR_E107A or LRR_W139C to the cultured cells exerted a lesser suppression on cell proliferation relative to WT LRR (Figure 5C,D). On Day 2, there was a significant difference in the rate of cell proliferation between cells treated with WT LRR and LRR_E107A or LRR_W139C. The results support the notion that interaction between LRRs and laminin-511 modulates cell proliferation.

### 2.5. Binding of LRRs to Laminin-511 Suppresses Renal Cystogenesis

**Figure 6:**
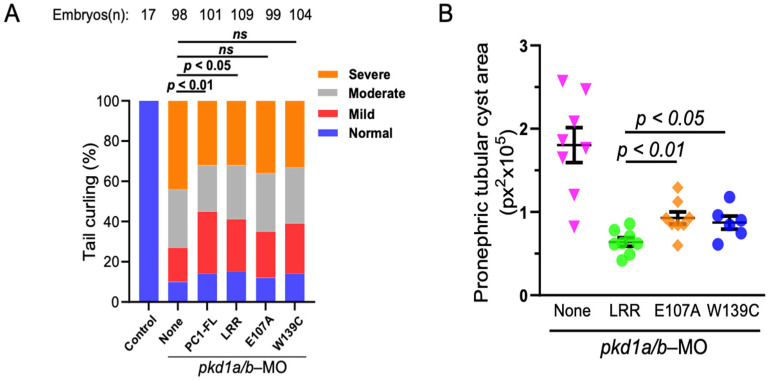
LRR–laminin interaction is necessary for anti-cystogenic effect of LRRs. (**A**) shows that transgenic expression of PKD1 or LRRs rescues tail curvature phenotype in pkd1-morphant ZF embryos, while both E107A and W139C-LRR mutants do not. Data presented are from at least 3 independent experiments. The total number (n) of embryos from combined multiple experiments is shown on top. Statistical analysis was performed by χ^2^ test. (**B**) Pronephric tubular cyst areas are quantitated in vehicle (None), LRR-, E107A-, or W139C-expressed ZF embryos co-injected with pkd1a/b morpholinos. Total cystic area is significantly reduced in LRR-expressed pkd1-MO ZF embryos but not with E107A and W139C. Cystic area is presented as pixels squared (px^2^). Data are presented as mean ± SEM. ns = not significant.

We next examine the role of LRR–laminin-511 interaction to ameliorate renal cystogenesis using pkd1a/b zebrafish morphants as described previously. mRNA expressing full-length PC1, WT LRR, or LRR mutants (E107A or W139C) were co-injected with pkd1a/b morpholinos. Dorsal-axis curvature and pronephric cystogenesis were analyzed at ~3 dpf (Figure 6). As reported earlier, injection of mRNAs expressing full-length PC1 or WT LRR (at 200 pg each) significantly suppressed dorsal-tail curvature in pkd1a/b morphants (Figure 6A). However, co-injecting LRR mutants, E107A, or W139C along with pkd1a/b morpholinos did not reverse tail curvature in ZF embryos. Likewise, total cystic area in pronephric tubules in pkd1a/b morphants was reduced by WT LRR but considerably less so by E107A or W139C LRR mutants (Figure 6B, Supplementary Figure S2). Thus, disruption of LRR–laminin interaction by mutation of critical residues in the LRR domain impairs the anti-cystogenic effect of LRRs.

## 3. Discussion

Mutations of PC1 account for the majority of ADPKD cases. The role of the ~3000-amino-acid-long N-terminal extracellular domain of PC1 (ECD) in disease pathogenesis is highlighted by the fact that many pathogenic variants in PKD1 with clinical significance lie in that region. Many studies have reported interaction of ECD of PC1 with extracellular matrix (ECM) [18,22]. Altered matrix integrity and anomalous expression of ECM proteins such as collagen, proteoglycans, focal adhesion protein tensin, laminin, and integrins have been noted in ADPKD [18,23]. Similarly, polycystic cells derived from PKD mice have been shown to have excessive adhesion to ECM proteins [24]. These abnormal cell–matrix interactions in PKD are believed to contribute to altered renal epithelial cell proliferation and cystogenesis.

In the current study, we explored the role of ECD of PC1 in the development of kidney cysts in ADPKD using a zebrafish model. Previous studies have shown that loss of pkd1a/b expression in zebrafish embryos leads to increased extracellular matrix deposition, resulting in dorsal-axis curvature in a dose-dependent fashion, and can be used as a quantitative readout [15]. In the current study, we found that the entire ECD is not capable of substituting the effect of full-length PC1 to reverse PC1-deficient phenotypes in zebrafish. Interestingly, the LRR domain in the absence of the rest of ECD is capable of reversing PC1-deficiency when it is overexpressed. The effects of LRRs are demonstrated by reversal of dorsal-axis curvature and pronephric cystic area, both well-characterized to represent PC1 function in zebrafish. The effect of LRRs acting from the extracellular space is supported by the finding that ER-targeted LRRs (LRR_KDEL, Figure 2C) have no effects. The observation that expression of truncated membrane-free ECD does not rescue PC1-deficient phenotypes whereas expression of full-length membranous-anchored PC1 has effect is likely because ECD has a much lower local effective concentration when expressed as an extracellularly secreted peptide. Since ECD is approximately 17 times longer than LRRs, the same dosage of injected mRNA will express LRRs in ~17-fold molar excess than ECD, thus underscoring that isolated LRRs are effective. Importantly, in the native state, the LRR domain is attached to PC1 anchored to the cell membrane. The local effective concentration of LRRs in the context of PC1 in the native tissue thus is likely much higher than when expressed as an isolated domain.

**Figure 7:**
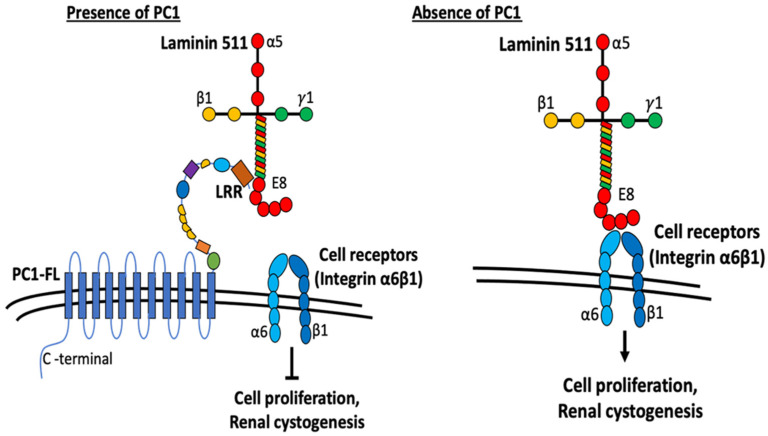
Working model depicting the role of LRRs in modulating cell proliferation and cystogenesis. Current work suggests the role of PC1 as a modulator of ECM components. N-terminal extracellular fragment of PC1 contains multiple domains similar to various adhesion proteins that are involved in cell–cell and cell–matrix contacts. LRR domains in PC1 interact with laminin-511 and inhibit its interaction with cell-surface receptors integrin α6ß1. Without PC1, laminin-511 interacts with integrin α6ß1 to trigger downstream signaling cascades promoting cell proliferation and renal cystogenesis. See text for more details.

A previous study by Malhas et al. reported that the LRR domain binds to extracellular matrix proteins including laminin, collagen, and fibronectin [17]. Laminins are extracellular matrix glycoproteins which make up a major component of the basement membrane. They are an important and biologically active part of the basal lamina on which cells sit that can influence cell differentiation, migration, and adhesion [25,26]. Laminins are heterotrimeric, consisting of three different chains, α, β, and γ. Many cell types (including HEK cells used in the study) secrete laminins when grown in cell culture; cell interaction with the secreted laminins affects cell behavior. To investigate the mechanism by which LRRs may be involved in anti-cystogenesis, we first demonstrate that extracellular application of LRRs inhibits proliferation of HEK cells which secrete laminin that consists of α5, β1, and γ1 chain (i.e., laminin-511, also known as laminin 10) [27]. We further demonstrate that LRRs bind to laminin-511 in vitro. Moreover, mutating key residues in the LRR domain important for binding laminin-511 diminishes its ability to inhibit HEK cell proliferation and to suppress pronephric cystogenesis in PC1-deficient zebrafish embryos. With respect to how LRR binding to laminin-511 affects cell proliferation, cells interact with laminin through cell membrane receptor integrins. HEK cells express endogenous integrin α6ß1, which is a known receptor for laminin-511 [28,29,30]. Binding to laminin through cell membrane integrin receptors activates EGFR tyrosine phosphorylation and MAPK phosphorylation to regulate cell proliferation [29]. Thus, LRRs may inhibit HEK cell proliferation by binding and competing laminin-511 interaction with integrin α6ß1 on the HEK cell membrane (Figure 7).

A similar mechanism likely explains how LRRs exert an anti-cystogenesis effect in the kidney (Figure 7). Laminin-511 is the predominant basement membrane component for renal tubules [31]. The LRR domain of PC1 in the basal membrane of renal tubular cells may modulate cell–matrix contact between tubular epithelial cells and the basement membrane to suppress intercellular events leading to cystogenesis including cell proliferation. In support of this hypothesis, laminin is shown to influence the identity of polycystic tubular epithelial cells [18]. Evidence also exists for alterations in laminin expression leads to PKD. Shannon et al. have shown that expression of truncated laminin α5 chain leads to cysts and renal failure in mice [32]. As above, integrins are cell-surface receptors for laminin. Integrin binding to laminin will activate intracellular signaling (see below) leading to cell proliferation. We propose that LRR binding to laminin would prevent its interaction with integrins and suppress proliferation and other processes of cystogenesis. In support for the notion, Lee et al. report that integrin signaling is upregulated in *Pkd1*-deleted mice, and deletion of integrin ameliorates cystogenesis and improves the renal function in these mice [33]. Thus, PC1 may act as an inhibitor of integrin-mediated signaling through regulating ECM–integrin crosstalk. Activation of integrin receptors leads to increased PI3K, Ca^2+^ level and Ras/Raf/Erk signaling pathways, all of which may be associated with ADPKD. Furthermore, PC1 colocalizes with integrin proteins in focal adhesions, cell–cell contact, and cilium in renal epithelial cells [18,34,35]. It is also reported that polycystic cells exhibit increased adhesion to laminin proteins through integrins [24], further supporting that PC1 exerts integrin–ECM interaction. Along the same line, laminin-332 and its binding partner integrin α6ß4 are altered in PKD [23]. Thus, LRRs of PC1 present in the basolateral membrane may regulate cell–matrix and cell–cell interactions through affecting cell-surface integrin receptors binding with laminin imbedded in the basement membrane or secreted between cells. Disturbances in these processes may alter cell proliferation, cell adhesion, and tissue architecture, which underlie the pathogenesis of ADPKD.

Finally, besides LRRs, other domains in the extracellular region of PC1 also bind proteins in the ECM and regulate cell–cell/matrix interaction. For instance, C-type lectin domain in the ECD of PC1 is shown to interact with Type I, II, and IV collagens as well as laminin proteins. It binds to carbohydrate residues in these proteins in a dose-dependent manner and may regulate cell–cell signaling [18]. Likewise, Ig-like PKD repeats in the ECD have strong homophilic interactions, and antibodies against these domains disrupt cell–cell contacts by reducing intercellular adhesion [22]. Our present study supports the view that altered ECM components by PC1 deficiency contributes to the pathogenesis of ADPKD. Other regions of PC1 undoubtedly also play important roles in the function of PC1 in anti-cystogenesis, such as the role of TM of PC1 forming channel complex with PC2 and the role of the C-terminal intracellular tail in regulating gene transcription as well as mitochondrial function [4,5,6,36,37].

## 4. Materials and Methods

### 4.1. Cell Culture and Transfection

HEK293 cells were grown in Dulbecco’s Modified Eagle Medium (Gibco, Grand Island, NY, USA), 10% fetal bovine serum (Gibco, Grand Island, NY, USA), 100 U/mL Pen Strep (Gibco, Grand Island, NY, USA), and 0.25 μg/mL Amphotericin B (Gibco, Grand Island, NY, USA) and maintained at 37 °C and 5% CO_2_. For plasmid transfections, Lipofectamine 2000 was used following manufacturer’s instructions (ThermoFisher Scientific, Waltham, MA, USA).

### 4.2. Plasmids, Site-Directed Mutagenesis, and In Vitro RNA Synthesis

pcDNA5-FRT-TO-PKD1-HA was generously provided by Dr. Julie Xia Zhou (University of Kansas Medical Center, Kansas City, KS, USA). Targeted deletions and substitutions were introduced by Q5^®^ Site-Directed Mutagenesis Kit (New England Biolabs, Ipswich, MA, USA) according to the protocol provided by the manufacturer and validated by sequencing. Bases corresponding to amino acids 3075–4303 and 177–4303 were deleted from the *PKD1* full-length cDNA sequence to create ECD- (1–3074 aa) and LRR- (1–176 a.a) expressing plasmids, respectively. Subsequently, capped mRNAs were synthesized from enzyme-digested linear constructs using HiScribe^TM^ T7 in Vitro Transcription Kit following the manufacturer’s instructions (New England Biolabs, Ipswich, MA, USA).

### 4.3. Zebrafish Housing and Morpholino (MO) Injection

Wild-type zebrafish (*Danio rerio*) were bred and maintained at 28 °C in a 14–10 h light–dark cycle. All procedures were followed in accordance with the approved protocols by the University of Iowa Animal care and Use committee. Experiments were performed as previously described [38]. Briefly, knockdown of zebrafish *pkd1a* and *pkd1b* genes was carried out by injecting previously validated gene-specific antisense MO oligonucleotides (Gene Tools, LLC, Philomath, OR, USA) [15]. At 1–4 cell stage, each fertilized egg was microinjected with 2 ng of either *pkd1a* or *pkd1b* MO or both. In addition to MOs, embryos were injected with 200 pg of in vitro synthesized capped mRNAs or vehicle in a total volume of 4.6 nL by using a Nanoject microinjector (Drummond Scientific, Broomall, PA, USA). Injected embryos were incubated in egg water at 28 °C and analyzed at 3 days post-fertilization (dpf). For histological sectioning and staining, embryos at 3 dpf were fixed in 4% paraformaldehyde at 4 °C overnight. Afterwards, embryos were sequentially treated with 10% and 20% sucrose for 30 min each and then with 30% sucrose until they sink to the bottom of the tube. Then, the embryos were fixed in a mold and embedded in optimal cutting temperature (OCT) medium and sectioned at 5 μm. Following Sullivan-Brown et al., tissue sections were stained in Hematoxylin and Eosin stain and imaged on a Nikon Eclipse E600 [39]. Afterwards, pronephric tubular cyst areas as marked by asterisks in the lower panel of Supplementary Figures S1 and S2 are measured from the procured images and presented as pixel square.

### 4.4. Structure Refinement, Molecular Modeling, and Interaction Analysis

The atomic coordinates of laminin-511 were retrieved from the Protein Data Bank (PDB ID: 5XAU). It consists of three chains, α5, β1, and γ1, which assemble into a cross-shaped heterotrimer [30]. For low-density regions or missing residues, the WT structure of laminin-511 was modeled by MODELER 9.20 embedded in the PyMOL plugin PyMod 2.0 (www.pymol.org, accessed on 16 January 2021), using 5XAU as a template. LRRs (24–176 aa) of PKD1 were used to generate a three-dimensional (3D) homology model from multiple threading alignments and iterative structural assembly simulations using I-TASSER [40,41]. I-TASSER homology modeling gives us five structures corresponding to the five largest structure clusters. The confidence of each model is quantitatively measured by its C-score (-5-2), where a model with a high C-score signifies a model with higher confidence and vice versa. We selected the LRR 3D atomic model with the highest C-score for our study. LRR WT structure was used to create E107A and W139C mutations using a mutagenesis plugin embedded in PyMOL. Mutants and WT structures of LRRs were energy minimized using SPDBV to remove high-energy configurations by changing their coordinate geometries in such a way as to release internal constraints and reduce the total potential energy. The final energy-minimized structures of laminin-511 and LRRs (WT, E107A, and W139C) were used for blind docking using ClusPro2 [42]. Protein–protein complexes produced by ClusPro2 are considered to have a high degree of confidence because it scored as the topmost protein–protein docking tool in the CAPRI assessment recently (https://cluspro.org/home.php, accessed on 16 January 2021).

### 4.5. Cloning, Expression, and Purification of LRRs

cDNA fragments encoding LRRs (24–176 aa) were amplified using PCR and subcloned into the pGEX-4T-1 vector to generate GST fusion proteins. The expression vector harboring point mutations for LRRs (E107A and W139C) was generated following the standard protocol of the Q5^®^ Site-Directed Mutagenesis Kit (E0554S, New England Biolabs, Ipswich, MA, USA). The LRR insert was confirmed by double digestion and DNA sequencing. Expression vectors were transformed and expressed into Escherichia coli BL21 DE3 cells. Cells were grown at 37 °C and induced with 0.5 mM IPTG (Sigma, Saint Louis, MO, USA) when absorbance reached 0.5–0.6 at 600 nm. Overnight-grown cell culture was centrifuged at 4000× *g* for 10 min at 4 °C, and pellets were dissolved in cell lysis buffer (50 mM Tris–HCl buffer, pH 8.0, 500 mM NaCl, 5% (*v*/*v*) glycerol, 2 mM DTT, 0.1 mg/mL lysozyme, 5 mM phenyl methane sulfonyl fluoride (PMSF), 1% (*v*/*v*) Triton X-100 (IBI Scientific Biochemical Corp., Dubuque, IA, USA), and 0.1% Tween-20). Sonication was used for cell lysis on ice for 10 min, and pellets (now called inclusion bodies; IBs) were collected after centrifugation for 30 min at 13,000 rpm at 4 °C. IBs were washed three times with milli-Q water to obtain pure IBs. Further, IBs were solubilized with 0.3% N-lauroylsarcosine (sarcosine) overnight at room temperature to obtain a high amount of our solubilized protein. To remove sarcosine, the sample was dialyzed overnight against buffer (50 mM Tris–HCl buffer, pH 8.0, 200 mM NaCl, and 1 mM DTT) at 4 °C. The supernatant collected after centrifugation was filtered with a 0.22 um filter before loading to a glutathione affinity column equilibrated with buffer (50 mM Tris–HCl buffer, pH 8.0, 200 mM NaCl, and 1 mM DTT) to capture the GST-tagged LRRs (GST-LRR). The column was then washed with wash buffer (50 mM Tris–HCl buffer, pH 8.0, 200 mM NaCl, and 1 mM DTT), and our target protein bound to resin was eluted in elution buffer in a stepwise manner, starting with wash buffer containing 5 mM, 10 mM, and 20 mM glutathione. The purified fractions were collected, concentrated, and the purity of the protein was assessed on SDS-PAGE (12%) under reducing conditions. The integrity of GST-LRR expression was further confirmed by thrombin treatment (Supplementary Figure S3). For the purification of GST-LRR mutants (E017A and W139C), we followed the same protocol as GST-LRR WT. All the purification steps were performed at 4°C unless otherwise stated.

### 4.6. Cell Proliferation Assays

The effects of GST-LRR on the proliferation efficiency of HEK cells were studied using the freshly purified GST-LRR at varying concentrations (10, 30, 100, and 300 ng/mL). HEK cells were grown in Dulbecco’s Modified Eagle Medium (DMEM) as described above. Cell proliferation efficiency was measured by an automatic cell counter (Bio-Rad, Hercules, CA, USA) for Days 0, 1, 2, and 3. The experiment was performed in a 24-well plate with complete (medium + 10% FBS) and incomplete media (medium only). To harvest the cells, the medium was aspirated, and cells were washed with PBS. Trypsin (0.05%, 200 μL, Gibco, Grand Island, NY, USA) was added in each target well and incubated it for 5 min at room temperature before adding complete DMEM (800 μL) to neutralize the trypsin. Cell counting was performed by mixing 20 μL of the harvested cells with 20 μL of trypan blue. The same protocol was followed for Days 0, 1, and 2 for the effects of E107A and W139C on HEK cells proliferation as well.

### 4.7. Protein Co-Purification Assay

Interaction between GST-LRR and laminin-511 (AMSBIO, Cambridge, MA, USA) was studied using co-purification assay as previously studied for several affinity-tagged proteins [43,44]. Purified GST-LRR WT was incubated with glutathione resins in binding buffer/wash buffer (50 mM Tris–HCl buffer, pH 8.0, 200 mM NaCl, and 1 mM DTT), and the resins were washed after a few minutes with the same buffer to remove the unbound GST-LRR WT. After washing, laminin-511 (~50 ug) was added to the affinity resin already bound with GST-LRR and incubated for 2 h at 4 °C. Subsequently, resins were washed twice with binding buffer to remove the non-specific interactions between the proteins, and finally, the complex was eluted with an elution buffer containing 20 mM reduced glutathione. We followed the same procedure for E107A and W139C mutants for this study as well.

### 4.8. Statistical Analysis

Descriptive data were presented as means ± SEM. All experiments were repeated at least three times. Prism 8 (GraphPad software, Version 10.2.0) and excel was used to analyze and present the data. Statistical comparisons between the two groups of data were made using two-tailed unpaired Student’s *t*-test. The χ^2^ test was used to analyze the statistical significance across groups in zebrafish morpholino studies by comparing the relative ratio of each category of curvature between groups. *p* < 0.05 was considered as statistically significant.

## 5. Conclusions

Current work illustrates the anti-cystogenic role of the LRR domain located in the N-terminus of PC1 in the zebrafish model system. The LRR domain is shown to interact with laminin-511 and may inhibit its interaction with cell-surface receptors such as integrin α6ß1. Loss of PC1 enhances interaction between laminin-511 and integrin α6ß1 that induces downstream signaling pathways promoting cell proliferation and renal cystogenesis. This warrants further investigation in mice models, and additional research is needed to uncover the underlying molecular mechanism. This will open new avenues for therapeutic development in ADPKD cure.

## Data Availability

The data that support the findings of this study are available on request from the corresponding author.

## Supplementary Materials

The following supporting information can be downloaded at: https://www.mdpi.com/article/10.3390/ijms25052886/s1.
