# Supplementary material for: Leucine-Rich Repeat in Polycystin-1 Suppresses Cystogenesis in a Zebrafish (Danio rerio) Model of Autosomal-Dominant Polycystic Kidney Disease
Source: Int J Mol Sci. 2024 Mar 1;25(5):2886. doi: 10.3390/ijms25052886 (PMC10932423; doi:10.3390/ijms25052886)
Supplement: Supplementary file 1 [file ijms-25-02886-s001.zip › ijms-2867412-supplementary.pdf]

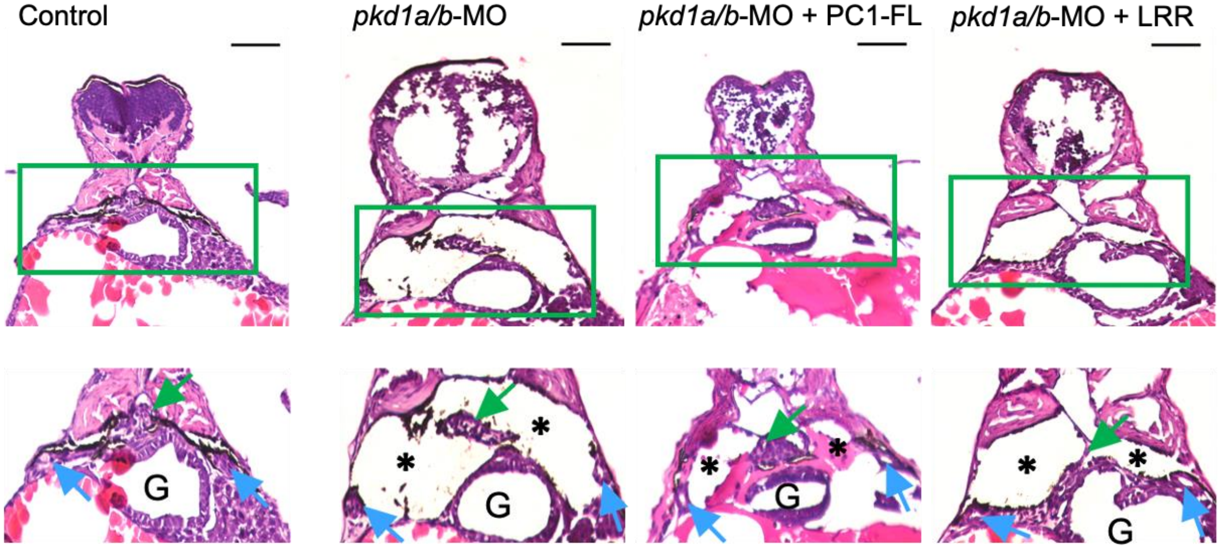


**Supplementary Figure S1. Transgenic expression of FL-PC1 or LRR domain (at 200pg each) suppresses pronephric tubular cysts in *pkd1a/b*-morphant embryos.** Upper panel presents H&E-stained cross-sections of ZF embryos depicting the structure of pronephros in control, *pkd1a/b*-MO coinjected with vehicle or mRNA expressing FL or LRR domain, respectively. Lower panels present the enlarged images of corresponding green boxes. Pronephric tubular cysts (asterisk), pronephric duct (blue arrows) and glomerulus (green arrows). Scale bars: 50 µm.

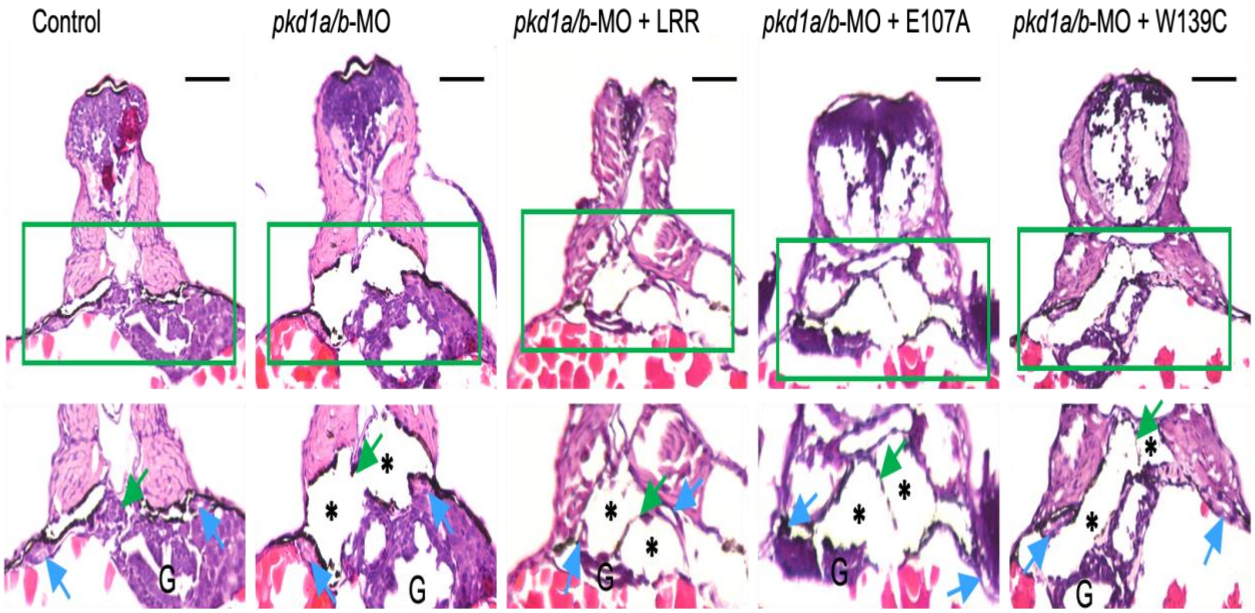


**Supplementary Figure S2. LRR-laminin interaction is necessary for anti-cystogenic effect of LRR.** Upper panel shows H&E-stained cross-sections of ZF embryos depicting the structure of pronephros in control embryos and pronephric tubular cysts in pkd1a/b-morphant ZF embryos coinjected with vehicle or mRNA expressing WT-LRR, E107A or W139C mutants. Lower panels present the enlarged images of corresponding green boxes. Pronephric tubular cysts (asterisk), pronephric duct (blue arrows) and glomerulus (green arrows). Scale bars: 50 µm.

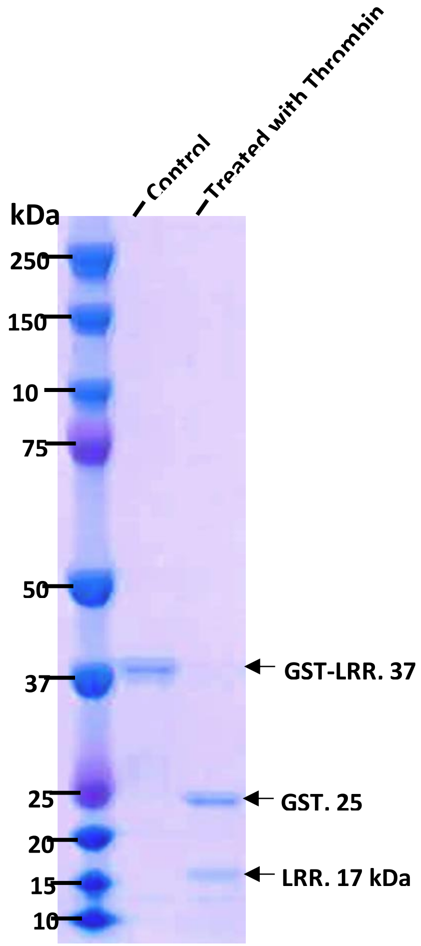


**Supplementary Figure S3.** The integrity of GST-LRR expression was confirmed by thrombin treatment.
